# The Cellular Response to Transcription-Blocking DNA Damage

**DOI:** 10.1016/j.tibs.2018.02.010

**Published:** 2018-05

**Authors:** Lea H. Gregersen, Jesper Q. Svejstrup

**Affiliations:** 1 Mechanisms of Transcription Laboratory, The Francis Crick Institute, 1 Midland Road, London NW1 1AT, UK

**Keywords:** DNA damage response, nucleotide excision repair, transcription-coupled nucleotide excision repair, Cockayne syndrome, UV-sensitivity syndrome, transcription restart

## Abstract

In response to transcription-blocking DNA lesions such as those generated by UV irradiation, cells activate a multipronged DNA damage response. This response encompasses repair of the lesions that stall RNA polymerase (RNAP) but also a poorly understood, genome-wide shutdown of transcription, even of genes that are not damaged. Over the past few years, a number of new results have shed light on this intriguing DNA damage response at the structural, biochemical, cell biological, and systems biology level. In this review we summarize the most important findings.

## Highlights

Biochemical and genetic studies indicate crosstalk between TC-NER factors and factors regulating RNAPII backtracking and elongation.

TC-NER is controlled by an unexpectedly complex layer of post-translational regulation targeting repair factors and RNAPII itself.

Dramatic changes to the transcriptional program in response to UV irradiation have been revealed by high-throughput genome-wide and time-resolved measurements.

Chromatin remodelers, splicing factors, noncoding RNAs, and RNA modifications provide a multifaceted transcriptional response to UV irradiation.

## The Transcription-Related DNA Damage Response

Cells respond to DNA damage in a variety of ways, including stalling of the cell cycle, initiation of DNA repair, and regulation of transcription, translation, and the ubiquitin–proteasome system to contend with the challenge. However, depending on the nature of the DNA lesion, cells react differently at the level of transcription. For example, double-stranded DNA (dsDNA) breaks appear to mostly result in localized interruption of transcription around the site of DNA breakage [1] while most base damage appears to be largely ignored, resulting mainly in temporary pausing of elongating RNAP (see, e.g. [2]). By contrast, DNA lesions such as those generated by UV irradiation or by chemical modification such as a platinum adduct not only form a blockade of ‘local’ RNAP progression when they occur in the transcribed strand, but also elicit global shutdown of transcription. Understanding how cells respond to such transcription-blocking lesions comprises one of the last important frontiers in DNA repair research.

## Transcription-Syndrome Group B Proteins Play Critical Roles in TC-NER

Transcription-coupled nucleotide excision repair (TC-NER) is unique in that it is initiated via the recognition of RNAP rather than by DNA lesions (see Table 1 for an overview of repair factors). Indeed, UV-induced and bulky lesions in the transcribed strand constitute a strong barrier to forward RNAP translocation and result in polymerase stalling/arrest 3, 4, 5, 6. The bulk of RNAP effectively shields the DNA lesion from recognition by DNA damage-binding factors 4, 7, explaining why RNAP, rather than the DNA lesion, is the focal point of TC-NER. Interestingly, the initial discovery of TC-NER was made in mammalian cells 8, 9 and only later extended to bacteria [10] and yeast [11]. While the molecular details of TC-NER are likely to vary somewhat between the different domains of life, there are also surely many similarities, and a description of the process in bacteria, yeast, and mammalian cells is therefore relevant to the building of working models for the process.

**Table 1:** NER and Transcription–Repair Coupling Factors

Mammals	Yeast	Bacteria (analogous)	Catalytic activity
**NER factors**			
XPA DNA damage verification and scaffold for recruitment of other NER factors	Rad14		
**TFIIH** Comprises two subcomplexes, a ‘core’ comprising six subunits (XPB, P62, P52, P44, P34, and P8) and a three-subunit kinase comprising CDK7, cyclin H, and MAT1; the core and kinase subcomplexes are bridged by the XPD subunit			
XPD (ERCC2) Promotes opening of DNA around the lesion; DNA damage verification	Rad3	UvrD Removes damaged ssDNA oligonucleotide; Can translocate RNAP backward for TC-NER UvrB Involved in DNA damage verification	5′–3′ helicase/ATPase/translocase
XPB (ERCC3) Promotes opening of DNA around the lesion	Rad25 (Ssl2)	UvrD	3′–5′ helicase/ATPase
TTDA, P8 (GTF2H5) Stimulates the ATPase activity of XPB	Tfb5		
P62 (GTF2H1)	Tfb1		
P52 (GTF2H4)	Tfb2		
P44 (GTF2H2)	SSL1		Ubiquitin ligase
P34 (GTF2H3)	Tfb4		
CDK7	Kin28		Kinase activity; the kinase complex of TFIIH is not required for NER
Cyclin H	Ccl1		
MAT1	Tfb3		

RPA Binds ssDNA revealed after unwinding	Rpa		
XPG Cleaves damaged strand downstream of lesion	Rad2	UvrC Cleaves damaged strand upstream and downstream of lesion	Endonuclease
XPF–ERCC1 Cleaves damaged strand upstream of lesion	Rad10–Rad1	UvrC Cleaves damaged strand upstream and downstream of lesion	Endonuclease
**TC-NER factors**			
CSB (ERCC6) Recognizes damage-stalled RNAPII on upstream side; promotes forward movement of RNAPII	Rad26	TRCF/Mfd Translocates RNAP forward; recruits NER factors through interaction with UvrA	Translocase
CSA (ERCC8) Part of the E3 ubiquitin ligase complex CLR4 Required for recruitment of UVSSA to CSB	Not found	Not found	
UVSSA Promotes stabilization of CSB	Not found	Not found	
USP7 Deubiquitylates CSB	Not found	Not found	Ubiquitin protease
**GG-NER factors**			
XPC Recognizes helix-distorting lesions	Rad4	UvrA? Recognizes DNA damage together with UvrB	
DDB1–DDB2 complex (XPE) Recognizes DNA lesion directly, kinks the DNA to provide recognition by XPC; also part of the CRL4 E3 ubiquitin ligase complex	Not found	Not found	
**Gap filling (DNA replication)**			
Proliferating cell nuclear antigen (PCNA) DNA clamp, processivity factor for DNA Pol	Pcna/Pol30		
Replication factor C (RFC) Clamp loader	Rfc		
DNA Pol δ/ε	DNA Pol δ/ε (Pol3, Pol2)	DNA Pol I	DNA Pol
DNA ligase 1	DNA ligase I (Cdc9)	Ligase	DNA ligase

## TC-NER in *Escherichia coli*

In bacterial NER, the **UvrA** (see Glossary) subunit of the UvrAB complex recognizes the DNA lesion. This is followed by subunit exchange, with UvrC replacing UvrA. UvrC then cleaves the DNA on each side of the lesion, generating an excision product that is removed by UvrD helicase, followed by gap filling and ligation [12]. Transcription intrinsically inhibits repair [13], but bacteria use **Mfd** (also known as **TRCF**) to perform TC-NER. Mfd is a DNA translocase that recognizes RNAP and translocates it forward, resulting in polymerase dissociation when its path is blocked by DNA damage [14]. Mfd then recruits the basic NER machinery through direct interaction with UvrA and repair of the damage proceeds as outlined above [15]. This was until recently believed to be the main or only way in which transcription-blocking lesions are removed in *E. coli*. Interestingly, a recent report by Nudler and colleagues indicates that UvrD can bind RNAP and use its ATPase activity to translocate the polymerase backward on DNA, exposing the DNA lesion and allowing the general NER machinery to gain access [16]. So, besides removing the incised DNA oligonucleotide after NER, UvrD may also contribute to an early step of TC-NER [17]. It may be relevant to mention that Selby and Sancar have questioned the importance of this new role of UvrD in bacterial TC-NER 18, 19.

In a broad sense, Mfd is the functional analog of human CSB and budding yeast Rad26, while the role of UvrD – at least in terms of removing the incised, lesion-containing oligonucleotide – is performed by **TFIIH** in eukaryotes. These eukaryotic proteins are described in more detail below.

## TC-NER in the Yeast *Saccharomyces cerevisiae*

Much of the progress in understanding TC-NER has been based on cellular assays, several of them developed in budding yeast. In these approaches, lesion removal in the transcribed strand (TS) is compared with that in the non-transcribed strand (NTS) at nucleotide resolution across a gene region [20]. Using such methods it has been shown that mutation of the gene encoding the TC-NER factor Rad26 specifically decreases the repair rate in the TS [21]. Interestingly, deletion of *RPB9*, encoding an RNAPII subunit, also affects TC-NER, and cells lacking both *RAD26* and *RPB9* are much more TC-NER defective and also UV sensitive (while the single mutants are not) [22], suggesting functional redundancy between RNAPII and Rad26 in TC-NER.

Somewhat surprisingly, *RAD26* is dispensable for TC-NER in certain contexts. In the absence of the gene encoding the conserved elongation factor **Spt4**, or with certain mutations in Spt5 (Spt4’s essential partner), TC-NER is restored in *rad26* cells 23, 24. Mutations in other genes encoding RNAPII subunits or elongation factors also restore TC-NER in *rad26* cells 22, 25. These data are important as they suggest that Rad26 is not required for the TC-NER reaction *per se* and underscore the important role played by the RNAPII elongation complex itself in determining repair efficiency.

A number of different models, which are not necessarily mutually exclusive, may explain the role of Rad26 in TC-NER. One possibility is that another protein can substitute for Rad26. In this respect it is interesting that yeast **Sen1** has recently been shown to play a role in TC-NER that is distinct from that of Rad26 [26]. Alternatively, efficient and processive transcriptional elongation may represent a particular threat to genome stability in the presence of transcription-blocking DNA damage, and Rad26 would in this model help prevent RNAPII elongation complexes reaching, or becoming irreversibly arrested at, such lesions. In this scenario transcription elongation efficiency is reduced when Spt4/Spt5 or certain other elongation factors are absent, thus eliminating the need for Rad26.

Interestingly, Xu *et al*. recently used a biochemical approach combined with cryoelectron microscopy to investigate Rad26 function [5]. Their structure reveals that Rad26 binds to DNA upstream of RNAPII, dramatically altering the DNA path. It also suggests that Rad26’s translocase activity promotes forward movement of RNAPII by pulling DNA toward itself while positioned on the upstream side of the polymerase. This does not result in appreciable dissociation of RNAPII, or read-through, at DNA damage. However, Rad26 can push RNAPII through other obstructions, such as a poly-A tract or a polyamide bound to DNA [5]. These findings confirm and greatly expand on earlier studies showing that the human Rad26 counterpart CSB also fails to dissociate RNAPII from DNA damage but can push the polymerase forward, toward the DNA lesion [6].

**Figure 1:**
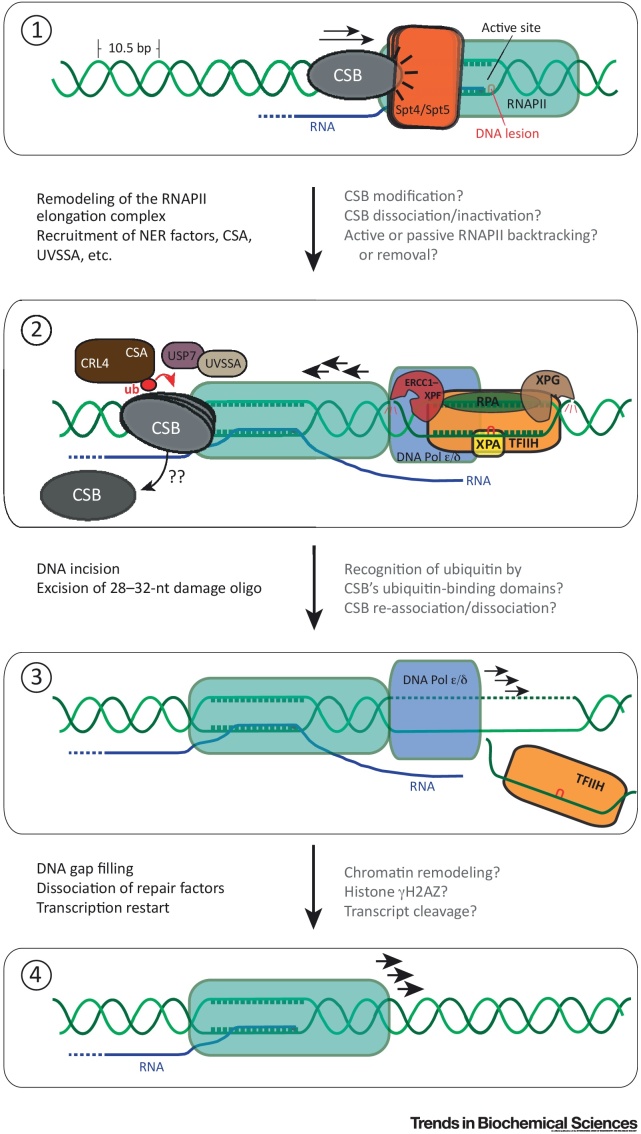
Models for Transcription-Coupled Nucleotide Excision Repair (TC-NER). (1) After RNAPII stalls at a transcription-blocking lesion, CSB is recruited. CSB’s precise role is unclear, but it may remodel the stalled RNAPII complex, for example to dislodge elongation factors such as Spt4/Spt5 (also called DSIF in humans). (2) To make space for NER, RNAPII must be either completely removed from DNA or backtracked. Importantly, if backtracking occurs it is likely to be by as much as 30–40 nucleotides due to the space requirements of RNAPII, repair factors, and the gap-filling DNA polymerase (DNA Pol) on DNA. Such backtracking might require CSB dissociation and could conceivably be facilitated by TFIIH. Repair factors (and probably DNA Pol) are recruited. DNA incisions are made by ERCC1–XPF and XPG and removal of lesion-containing ssDNA oligonucleotides is performed by TFIIH. (3) DNA gap filling, dissociation of NER factors, and marking of chromatin with γH2AZ. (4) Transcript cleavage and forward translocation, probably stimulated by factors such as ELL. A number of unanswered issues about the molecular details of the process are listed on the right. Note that the DNA characteristics of RNAPII and NER (footprint, melted DNA, excised oligo, etc.) are drawn to scale where possible. However, nucleosomes, as well as the dramatic bending of DNA in the elongation complex, are omitted for simplicity.

Somewhat frustratingly, these new data do little to explain how forward RNAPII movement might be helpful for TC-NER. Interestingly, however, inspection of recent structures of the RNAPII–DSIF (Spt4/Spt5) complexes 27, 28 suggests that the Rad26–RNAPII interaction (and thus presumably the CSB–RNAPII interaction) is incompatible with concomitant RNAPII–Spt4/Spt5 interaction. It is thus an intriguing possibility that Rad26 (CSB) may use its DNA translocase activity to move close to RNAPII, dislodging Spt4/Spt5 from the immovable, damage-stalled polymerase (Figure 1). As Spt4/Spt5 normally stabilizes the elongation complex to favor forward translocation, such displacement (and subsequent Rad26/CSB dissociation) might allow the RNAPII elongation complex to backtrack away from the lesion to allow subsequent repair factor recruitment and DNA repair, potentially helping explain why Rad26 is dispensable for TC-NER in cells lacking Spt4/Spt5 function.

## TC-NER in Mammalian Cells

Although the basic principles of TC-NER are likely to be conserved from yeast (and bacteria) to mammalian cells, it is clear from the factor requirements that there are notable differences (Table 1). For example, **Cockayne syndrome** A (CSA) and **UVSSA** are required for the process in human cells 29, 30, 31, 32, 33 but do not have obvious functional homologs in yeast or bacteria. Likewise, CSB has a functionally important ubiquitin-binding domain (UBD) that appears to be absent in Rad26 [34]. Moreover, as outlined above, and in contrast to prokaryotic Mfd, eukaryotic CSB and Rad26 are unable to dislodge damage-stalled RNAPII from DNA 5, 6, suggesting that the polymerase is either removed from DNA by other means or backtracked a significant distance away from the damage so that DNA repair and subsequent gap filling can occur. Interestingly in this context, TC-NER reconstitution experiments showed that an RNAPII-covered DNA lesion can be repaired in a CSB-stimulated manner and that RNAPII may be displaced from the DNA in an ATP-dependent but CSB-independent manner, likely by TFIIH [35]. TC-NER in these reconstitution assays occurred in the absence of CSA and UVSAA and thus they do not precisely reflect the factor requirements *in vivo*, but they nevertheless expose important, basic properties of the reaction. These results also potentially take on a new significance in light of the abovementioned data indicating that bacterial TC-NER analogously requires ATP- and UvrD-stimulated removal of RNAPII from the site of the DNA lesion so that the damage can be accessed [16]. An outline model for mammalian TC-NER is presented in Figure 1.

**Figure 2:**
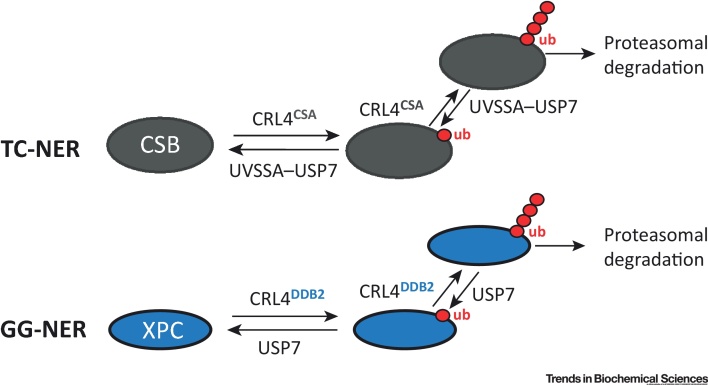
Analogous Regulation of Transcription-Coupled Nucleotide Excision Repair (TC-NER) and General Genome Repair (GG-NER) by Ubiquitin. CSB and XPC are specific for TC-NER and GG-NER, respectively, yet analogous ubiquitin pathways regulate their function. The CRL4 ubiquitin ligase complex is required for their ubiquitylation but uses CSA and DDB2, respectively, as targeting subunits. Unsurprisingly, CSA and DDB2 are also known to be specifically required for TC-NER and GG-NER, respectively. Likewise, USP7 is required for deubiquitylation of both, which in the case of CSB is likely to occur through its interaction with a targeting subunit, UVSSA. Abbreviation: ub, ubiquitin. See text for details.

Although the importance of CSA for TC-NER has been known for decades, insight into its precise function is limited. CSA is the substrate-recognition component of an E3 ubiquitin ligase complex, named **CRL4**, that can also use DDB2 or other proteins as separate substrate-recognition subunits [36]. One function of the CRL4^DDB2^ complex is to ubiquitylate XPC upon UV-irradiation. XPC is important for general genome repair (GG-NER) but not for TC-NER (Box 1). It is recruited to DNA damage by the DDB1–DDB2 complex, and XPC function and turnover at such sites is regulated at least partly via CRL4^DDB2^-mediated ubiquitylation [37]. It is potentially telling that, while DDB2 affects GG-NER only, its counterpart CSA is specifically required for TC-NER and is functionally tightly connected to CSB. Indeed, CSB is ubiquitylated in a CRL4^CSA^-dependent manner [38]. CUL4^DDB2^–XPC and CUL4^CSA^–CSB may thus represent functionally related enzyme–substrate constellations required for GG-NER and TC-NER, respectively. Both XPC and CSB also appear to be deubiquitylated by **USP7** 30, 39, 40 (see below) and regulated by SUMOylation 37, 41, further underscoring the analogies (Figure 2).

Box 1 **Mammalian NER Factors**

In GG-NER [87], the DNA lesion is recognized by the DDB1–DDB2 complex and XPC. The DDB1–DDB2 complex (DDB2 is also known as XPE) binds directly to the UV-induced lesion and stimulates the association of XPC, which in turn binds to DNA opposite the lesion causing local helix distortion. This provides a substrate for TFIIH binding and lesion verification. The two TFIIH helicase subunits XPB and XPD are required to simulate the opening of the DNA around the lesion. By contrast, in TC-NER [87] the lesion is recognized indirectly by CSB binding to stalled RNAPII elongation complexes (Figure 1). CSA, UVSSA, and USP7 promote ubiquitination and deubiquitylation of CSB, respectively, to regulate the reaction. The mechanisms of DNA incision and subsequent gap filling are the same in GG-NER and TC-NER [87]. TFIIH promotes the binding of XPA to ssDNA–dsDNA junctions. XPA is involved in damage verification and serves as a scaffold for NER factors. RPA binds the exposed ssDNA. The structure-specific endonuclease XPF–ERCC1 heterodimer (which cuts 5′ of the lesion) and XPG (which cuts 3′ of the lesion) are recruited by XPA. TFIIH binds and displaces the excised ssDNA oligonucleotide containing the damage and the ssDNA gap of 22–30 nt is then filled by DNA Pol δ or DNA Pol ε and sealed by DNA ligase 1.

Alt-text: Box 1

CSB contains a UBD. Without this UBD, CSB can still be recruited to DNA damage and is capable of assembling a TC-NER complex, but no DNA incision/repair occurs [34]. The precise molecular function of CSB’s UBD remains unclear, but one possibility is that it recognizes a ubiquitin moiety added by CRL4^CSA^ in either CSB or another protein. Interestingly, CSB is normally very dynamically associated with DNA damage sites but CSB lacking the UBD is slow to dissociate from such lesions [34]. So, it is possible that CRL4^CSA^-mediated ubiquitylation, and recognition thereof by CSB’s UBD, serves to somehow regulate activity and turn over CSB molecules around sites of RNAPII damage stalling, in a fashion analogous to that suggested for XPC 37, 42.

Interestingly in this connection, CSA is required for the recruitment of UVSSA to CSB after DNA damage [40] and UVSSA appears to be required for CSB stability 29, 30, 31, 40. In the absence of UVSSA, or its interaction partner USP7 30, 40, CSB becomes markedly degraded upon UV-irradiation. The loss of CSB seems unlikely to underlie the failure of UVSAA-deficient cells to perform TC-NER, as CSB overexpression cannot suppress the effect [30]. Importantly, the ubiquitin protease USP7 might work with UVSSA to deubiquitylate CSB and suppress its degradation. It is tempting to speculate that CSB ubiquitylation has not evolved to bring about its proteolysis but rather to regulate its function. In this scenario, dynamic, spatiotemporal (mono)ubiquitylation/deubiquitylation of CSB would be required to activate/inactivate or regulate the association/dissociation of the protein through the multistep TC-NER process (Figure 1). Conceivably, an early step in this process could require ubiquitylation while a later one requires deubiquitylation of the same, engaged CSB protein.

## Degradation of RNAPII

DNA damage-induced ubiquitylation occurs not only on CSB but also on a large number of other proteins, including RNAPII, which is modified at numerous sites in the largest subunits 43, 44, 45. At least some of these ubiquitylation events result in RNAPII degradation, which is likely to be a last-resort mechanism when TC-NER fails. The details of the last-resort mechanism have recently been reviewed [46] and are not outlined in detail here. It is, however, important to underscore that there is a very close connection between transcription, TC-NER, and RNAPII ubiquitylation. Thus, defects in the TC-NER pathway or in transcription can have significant consequences for RNAPII ubiquitylation and degradation [47]. While CSA (CRL4^CSA^) is not directly involved in RNAPII ubiquitylation/degradation, cells lacking CSA (or CSB) nevertheless have defects in RPB1 degradation [48], at least partly because the transcription response to DNA damage is altered in these cells [49]. Moreover, while RNAPII degradation is not required for TC-NER [46], it is not inconceivable that RNAPII ubiquitylation (perhaps as a non-degradative signaling event) does play a role in the process. Finally, RNAPII ubiquitylation (and degradation) can occur in response to a number of events that halt transcript elongation [46], making it imperative to explore the mechanism in detail before making conclusions on causative connections between DNA damage, DNA repair/transcription factors, and RNAPII ubiquitylation.

## CSB in Transcriptional Regulation and Disease

CSB deficiency results in lack of TC-NER and in Cockayne syndrome (CS), but this does not signify that TC-NER deficiency underlies CS a severe neurological disorder [50] (Box 2). If a causal relationship existed, mutation of the basal NER genes (most of which are also absolutely required for TC-NER) would be expected to give rise to the same severe disease. Instead, deficiency in these factors gives rise to **xeroderma pigmentosum (XP)**, a relatively milder disease, with a high incidence of skin cancer. Likewise, UVSSA is required for TC-NER (but not GG-NER), yet mutations in this gene give rise to an even milder disease, UV-sensitivity syndrome (UV^S^S) 29, 31.

Box 2 **Syndromes Associated with Defects in GG-NER and TC-NER**

**XP**

XP is caused by general defects in NER, with seven complementation groups (XPA–G; Table 1) and an XPV variant caused by defective translesion DNA synthesis. XP patients are extremely photosensitive and have a 1000-fold increase in susceptibility to skin and eye cancer. In addition, exposure to sun leads to freckle-like pigmentation in all XP patients. The severity of the symptoms varies between patients and around a third of XP patients also display neurological deficiencies, characterized by microcephaly, hearing loss, and progressive cognitive impairment that is likely to be caused by transcription deficiencies [88].

**CS**

CS patients are characterized by short stature, wrinkled skin, sunken eyes, large ears, a pointed nose, and prematurely thin and grey hair. Although CS is first and foremost a neurodevelopmemental disorder, patients also suffer from severe, progressive neurological degeneration. Neurological symptoms include hearing loss, cataracts, retinal dystrophy, mental retardation, and progressive ataxia. CS patients are photosensitive but do not develop marked pigmentation defects and do not have an increased frequency of skin cancer. Some mutations, in XPB, XPD, or XPG, result in combined XP–CS. See main text for details.

**Trichothiodystrophy (TTD)**

TTD is characterized by sulfur deficiency, brittle hair and nails, short stature, and intellectual impairment. Approximately half of TTD patients are photosensitive, but they do not develop pigmentation defects and do not have the predisposition to skin cancer observed in XP patients. Some TTD patients also have neurological symptoms, including microcephaly and dysmyelination. TTD is caused by mutations in the two helicases XPB and XPD and in GTF2H5 (TTDA), which are all subunits of TFIIH (see Table 1 in main text). TTD mutations interfere with TFIIH’s function in transcription 88, 89, which is likely to explain the different clinical outcome compared with XP.

**UV**^**S**^**S**

UV^S^S patients are photosensitive but do not display any of the developmental symptoms present in CS and some XP patients. UV^S^S is caused by a lack of functional UVSSA, as well as specific mutations in CSA and CSB. UV^S^S is likely to be caused by impaired TC-NER. The milder UV^S^S symptoms compared with CS are likely to be explained by the additional involvement of the CS protein in transcription regulation. It is unclear why certain mutations in CSA and CSB lead to UV^S^S while others result in CS, but these might affect TC-NER more than transcription.

Alt-text: Box 2

It is important to remember that CSB is part of the **SWI/SNF** family of ATP-dependent chromatin remodelers [51]. Chromatin remodeling by purified CSB was demonstrated *in vitro* long ago [52], but it has only recently been shown that CSB also affects chromatin structure *in vivo* [53]. This may help explain why CSB-deficient cells show altered expression at thousands of genes, also in the absence of DNA damage 54, 55, 56. A striking molecular hallmark of CS versus XP is the marked change in gene expression rather than DNA repair, as also evidenced by the dramatic consequences for transcription of *XPD* mutations that result in combined CS/XP disease compared with others in the same gene that result only in XP [56]. Importantly, CSB affects the expression of an unexpectedly large number of neuronal genes. Cellular reprogramming of CS fibroblasts to cells with neuron-like features is defective and neuroblastoma cells depleted for CSB show defects in neuronal gene expression and fail to differentiate and extend neurites 54, 57. Crucially, CSB function can be partially bypassed by overexpression of specific CSB target genes, most notably **SYT9** [58], which controls the release of neurotrophins such as **BDNF**, which is in turn important for neuronal differentiation and synaptic modulation. Strikingly, addition of BDNF, or pharmacological mimics such as amitriptyline, can compensate for CSB deficiency during neuronal differentiation, and SYT9 and BDNF are downregulated in CS patient brain tissue [58], indicating that suboptimal induction of neurotrophin-regulated gene expression programs, rather than DNA repair deficiency, underlies most neurological defects in CS.

It is worth underscoring, however, that CS is a complex disorder and it is thus likely that some of its other symptoms are caused by TC-NER defects, just as it has been argued that defects in mitochondrial biology and oxidative DNA damage repair may also play a role [59].

## The Transcription Response to UV Irradiation

UV-induced DNA damage in mammalian cells triggers the activation of some transcription factors and increases the half-life of p53 [60]. However, although individual genes are consequently highly upregulated by DNA damage, transcription across the rest of the genome is essentially shut down, as measured by a dramatic general reduction in the level of newly synthesized RNA 45, 61, 62, 63. Reduced RNA synthesis is sustained for several hours and normal transcription levels are fully restored only 24–48 h after UV exposure 64, 65. One of the identifying characteristics of CS cells is their effect on transcription restart: these cells are unable to recover significant gene expression after DNA damage [63].

Genome-wide measurements of repair kinetics indicate that (6-4) photoproducts are generally repaired quickly (within 4 h) while cyclobutane pyrimidine dimers (CPDs) require 12–48 h for complete removal, even in transcribed regions [66]. At first glance this could indicate that transcription shutdown is merely a consequence of DNA lesions blocking RNAPII progression and that restart occurs when repair has been completed. However, several lines of evidence indicate that shutdown is not simply a consequence of the physical blocking of RNAPII progress by DNA lesions but that it must also be mediated by signalling *in trans*. Extracts from UV-irradiated human cells have little transcription activity *in vitro* even on undamaged DNA templates [61] and transfection of an undamaged gene reporter into UV-irradiated cells fails to show significant expression levels until transcription restart occurs in endogenous genes as well [62]. Recent data indicate that merely the transfection of a UV-irradiated plasmid into undamaged cells is sufficient to elicit at least some aspects of the DNA damage response; namely, those related to co-transcriptional mRNA splicing [67].

**Figure 3:**
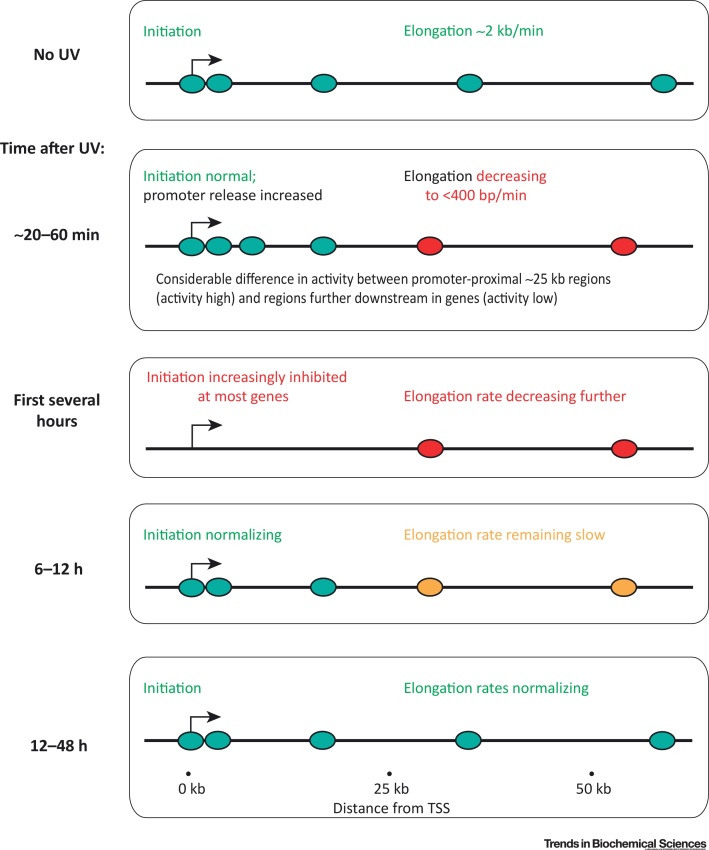
The Global Transcriptional Response to UV Irradiation. The different stages of transcriptional shutdown and restart in an average ‘long’ gene. Short genes (<20 kb) are often much less affected. See text for details.

Recent work shows that transcription shutdown and restart occur in several phases (Figure 3), with transcript elongation slowing already within 10–25 min of UV irradiation while transcriptional initiation is also inhibited shortly afterward [65]. This coincides with the release of promoter-proximal RNAPII 65, 68 and almost complete, global loss of the hypophosphorylated form of RNAPII, which is likely to be due to rapid hyperphosphorylation 61, 69. Transcriptional initiation recovers more quickly than elongation, which continues to be slow for many hours 61, 64, 65, 70. Moreover, transcription appears to be ‘spatially restricted’ for long periods, with the promoter-proximal 20–25 kb showing much more activity than the areas further downstream in genes after DNA damage 64, 65, 68 (Figure 3). This may help explain the striking observation that virtually all genes induced by DNA damage are short [71]: only short genes can be highly expressed after UV irradiation. It has been suggested that the release of RNAPII into the gene body might serve as a ‘sensor’ of DNA lesions [68], in that the shift from promoter-proximally paused to slowly elongating RNAPII might allow the TC-NER machinery to rapidly recognize and signal DNA damage when polymerases stall there.

Unsurprisingly, the dramatic change in transcript elongation after UV irradiation has consequences for co-transcriptional RNA-processing reactions such as splicing and termination. For example, UV irradiation leads to increased inclusion of certain ‘weak’ cassette exons, at least partly due to a decrease in elongation rates [72]. However, recent results indicate that splicing is also affected by depletion of certain **snRNP** spliceosomal proteins from chromatin following UV-induced damage. The mechanism underlying such displacement remains unclear, but it requires active transcription, is specific for transcription-blocking lesions, and is enhanced by the checkpoint kinase **ATM** [73]. Examples of UV- and ATM-dependent mRNA splicing are observed and, curiously, ATM is itself activated by **R loops**. The combination of transcriptional elongation inhibition, R loops, and ATM activation was proposed to form a positive feedback loop that triggers loss of specific spliceosomal proteins and thus a change in mRNA splicing on UV irradiation [73]. Intriguingly, another recent report shows that UV-induced CPDs can also result in the activation of **ATR**-dependent mechanisms, which likewise feed into mRNA splicing [67]. Thus, transcription-obstructing DNA damage elicits a complex cellular response that affects cassette exon splicing, at least partly via DNA damage checkpoint kinases 67, 73.

The dramatic changes in transcript elongation and spatial restriction on transcription also result in a marked change in mRNA polyadenylation site selection and transcriptional termination after UV irradiation [65]. This is expressed as a change in alternative last exon (ALE) splicing and results in examples of upregulation of much shorter transcript isoforms, which may at least in some cases function as stable RNAs. The involvement of RNA in the DNA damage response is covered in more detail below.

## Factors Affecting Genome-Wide Transcription Changes

Several factors involved in chromatin remodeling have been implicated in the shutdown of transcription after UV irradiation. For example, the **histone deacetylases (HDACs)** SIRT1 and HDAC1 are recruited to several damage-repressed genes 56, 74. Likewise, changes in RNAPII phosphorylation [61] and DNA binding by **TBP** [75] have been proposed to play a role. More recent evidence suggests that the sequence-specific transcription factor **ATF3** is also involved. ATF3 is a transcriptional repressor that is upregulated by DNA damage and that binds to the promoter region of a sizeable fraction of genes repressed by DNA damage (i.e., hundreds of genes) [74]. ATF3 recruits HDACs to repress transcription locally. One important role for CSB outside TC-NER is to counteract the repressive function of ATF3. ATF3-repressed genes such as DHFR take on the characteristics of facultative heterochromatin in CSB-deficient cells [56] and this can be at least partly counteracted by ATF3 depletion [74].

While ATF3 is clearly important for the repression of specific target genes after UV irradiation, the mechanism underlying repression of the rest of the genome is less clear. Recently, it was reported that the E3 ubiquitin ligase **UBR5** mediates transcriptional repression in response to UV downstream of the **PRC1** component BMI1 [76]. One possible target of the UBR5 E3 ligase was proposed to be the SPT16 component of the **FACT** complex, a histone chaperone [76]. However, this pathway for damage-induced transcriptional repression requires further investigation.

Unsurprisingly, a number of chromatin factors promoting efficient transcription have been implicated in allowing transcriptional restart after DNA damage. For example, the histone methyltransferase **DOT1L** [77] and the histone chaperones FACT [78] and **HIRA** [79] affect the ability of cells to restart transcription after UV irradiation. Moreover, the elongation factor **ELL** has also been shown to play a role [80]. Deficiency in any factor required for normal restart of transcription after UV irradiation would be expected to result in damage sensitivity; surprisingly, however, while cells with knockdown of DOT1L, FACT, and ELL display UV sensitivity, cells with robust HIRA knockdown do not [79].

## The Role of RNA in the Transcription-Related DNA Damage Response

While UV irradiation affects RNA processing, a number of recent papers have shown that RNA pathways in turn affect the cellular response to DNA damage. For example, UV-induced changes in ALE splicing result in upregulation of an RNA isoform, expressed from the ***ASCC3*** gene, that acts as a long noncoding RNA (lncRNA) rather than a protein-coding mRNA [65]. Interestingly, two distinct ASCC3 isoforms have opposite effects on transcription after DNA damage. The protein-coding ASCC3 mRNA encodes the 250-kDa helicase component of the ASC complex [81]. Upon knockdown of ASCC3 or other ASC complex members, cells show elevated levels of transcription 20 h after UV irradiation. By contrast, knockdown (or CRISPR knockout) of the alternative ASCC3 lncRNA transcript, which is normally upregulated by UV irradiation, has the opposite effect: this lncRNA is required to allow normal restart of transcription [65]. Intriguingly, the two ASCC3 isoforms crosstalk: in the absence of the mRNA isoform, the requirement for the lncRNA isoform is markedly reduced. The biochemical mechanisms underlying the functions of the different ASCC3 isoforms remain to be investigated.

Beyond lncRNAs, a role for small noncoding RNA, or the proteins connected to it, has been suggested by the finding that proteins involved in **miRNA** processing also impact the response to UV irradiation 82, 83, 84. Depletion of either Drosha or its interacting partner DGCR8 thus results in UV sensitivity. UV-induced phosphorylation of DGCR8 at serine_153_ is required for the removal of DNA lesions and restart of transcription after UV irradiation [82]. Surprisingly, DGCR8’s RNA-binding, and its Drosha interaction domains, are not required for this role, indicating that DGCR8’s function in the UV response is distinct from that in the miRNA pathway with Drosha. Likewise, an miRNA-independent function has been reported for Dicer to promote chromatin condensation in response to UV irradiation [84]. It remains unclear which RNA species, if any, are important for this response. The recruitment of Dicer to chromatin after UV irradiation is dependent on RNA, whereas the RNA-binding domains of DGCR8 are not required for DGCR8 function in this pathway [82]. The mechanisms underlying the intriguing UV sensitivity observed following Dicer, Drosha, and Ago2 depletion remain to be addressed.

Finally, a recent study found that RNA is extremely rapidly **m^6^A** methylated (within 2 min) by **METTL3** in response to UV irradiation [85]. Remarkably, de-methylation by **FTO** is also rapid, so that m^6^A methylation levels have already returned to normal by 8 min after UV irradiation. Lack of METTL3 nevertheless results in delayed DNA repair, slow transcription restart, and UV sensitivity. UV-induced m^6^A RNA modification does not affect the recruitment of typical NER factors such as XPA and TFIIH, but results in recruitment of DNA polymerase κ (Pol κ) to DNA damage sites. Such recruitment is physiologically important as Pol κ overexpression is sufficient to suppress the effects of METTL3 depletion. Pol κ has previously been shown to play a role in NER, with its recruitment ∼30 min after UV irradiation being dependent on XPA [86]. By contrast, the almost immediate m^6^A methylation-dependent recruitment of Pol κ to lesions is XPA independent. The biochemical mechanisms requiring fast Pol κ recruitment remain unclear, but one possibility is that it allows lesion bypass at genes that are being transcribed at the time of DNA damage induction (i.e., they possess nascent RNA for modification by METTL3 in the vicinity of the replicating DNA Pol), so that the replication machinery can clear such damage and allow rapid repair (by TC-NER, presumably). In any case, the two-stage involvement of Pol κ in the response to UV-induced DNA damage deserves further investigation.

## Concluding Remarks and Future Perspectives

The molecular mechanism of TC-NER has been elusive for several decades, but it seems a fair bet that most, if not all, of the factors required for it have now been uncovered. Therefore, a future challenge is to uncover the precise schedule of events required to remove a transcription-blocking lesion. How is RNAPII moved out of the way for DNA repair? What is the precise role of CSB? Is it required only to prepare the affected area (i.e., the chromatin, the lesion-stalled RNAPII, and/or the proteins associated with it) for the subsequent repair process or is CSB also actively involved in the repair reaction itself? What is the molecular role of CSA-mediated ubiquitylation/deubiquitylation and of CSB’s UBD? These are key questions that may require biochemical reconstitution or the development of new tools for *in vivo* discovery (see Outstanding Questions). Likewise, what are the mechanisms and factors responsible for the dramatic shutdown of transcription genome wide after UV irradiation? How is repression activated and then inactivated to allow transcription to recover? Finally, a fascinating and unexpected role for stable RNAs in the global regulation of transcription after DNA damage has been uncovered. Some of the processes and factors have been identified, but what is their precise mechanism of action? The next few years are certain to bring new exciting findings in this rapidly evolving field.

Outstanding Questions How is the stalled elongation complex recognized by CSB and the TC-NER machinery?

Can elongation complexes stalled at DNA lesions be distinguished from stalled RNAPII elongation complexes at non-lesion sites and if so, how?

Does ‘remodeling’ of RNAPII elongation complexes occur after UV irradiation? If so, is it necessary to remove elongation factors before TC-NER? Do elongation factors counteract TC-NER in mammalian cells as in yeast? Is the elongation complex merely backtracked away from the lesion or is it taken off the DNA altogether?

What is the functional role of CSB ubiquitination? Does it allow spatial and temporal regulation of CSB protein turnover or activity when associated with stalled RNAPII complexes? What is the precise role of CSA and UVSSA?

Which factors are required for global transcription shutdown? How are they activated by DNA damage? What it the signal that operates *in trans* to slow transcriptional elongation globally and how is it spread and restricted?

What is the role of the ASCC3 lncRNA isoform? Do the multiple other short transcript isoforms that are preferentially expressed after UV irradiation also play functional roles in the DNA damage response?

What is the role in the DNA damage response of short RNA species and the protein machinery that processes them?

## Glossary

**ASCC (activating signal cointegrator 1 complex)**: poorly understood factor comprising ASCC1, ASCC2, ASCC3, and TRIP4.
**ATF3 (activating transcription factor 3)**: a sequence-specific transcriptional repressor.
**ATM (ataxia telangiectasia mutated)** and **ATR (ataxia telangiectasia and Rad3-related)**: checkpoint kinases that are important for the cellular response to DNA damage.
**BDNF (brain-derived neurotrophic factor)**: a neurotrophin/growth factor that specifies neuronal identity. BDNF and amitriptyline, its pharmacological mimic, bind to cell-surface receptors to elicit cell signaling.
**Cockayne syndrome (CS)**: severe human syndrome mainly caused by mutations in CSA or CSB. The budding yeast homolog of CSB is Rad26.
**CRL4 (Cullin-ring E3 ubiquitin ligase)**: comprising CUL4A/B, DDB1, and RBX1; uses proteins such as DDB2 and CSA as its substrate-specifying subunits.
**DOT1L (disruptor of telomeric silencing 1-like)**: histone methyltransferase with activity toward histone H3 lysine 79.
**ELL (elongation factor for RNAPII)**: suppresses transient pausing by polymerase.
**FACT (facilitates chromatin transcription)**: a histone chaperone involved in transcription and DNA replication and repair.
**FTO (fat mass and obesity associated)**: an RNA demethylase.
**HIRA (histone cell cycle regulator)**: a histone chaperone.
**Histone deacetylases (HDACs)**: proteins that deacetylate histone; includes factors such as HDAC1 and SIRT1.
**m^6^A (N^6^-methyladenosine)**: a common methylation event in mRNA.
**METTL3 (methyltransferase-like 3)**: responsible for methylation of adenosine at position N6.
**Mfd (mutation frequency decline)** also known as **TRCF (transcription–repair coupling factor)**: represents the bacterial analog of human CSB and budding yeast Rad26.
**miRNA (microRNA)**: small noncoding RNA molecules (around 22 nucleotides) found in plants, animals, and some viruses that function in RNA silencing and post-transcriptional regulation of gene expression.
**PRC1 (polycomb repressive complex 1)**: transcriptional repressor complex containing subunits such as BMI1.
**R loop**: a three-stranded nucleic acid structure in genes comprising a DNA:RNA hybrid and the associated non-template single-stranded DNA (ssDNA).
**Sen1 (splicing endonuclease 1)**: an ATPase–helicase; homolog of the human senataxin protein.
**snRNPs (small nuclear ribonucleoproteins)**: RNA–protein complexes of at least five different kinds (U1, U2, U4, U5, and U6) that combine with RNA and other proteins to form a spliceosome, responsible for splicing of pre-mRNA.
**Spt4/Spt5 (transcription elongation factor)**: conserved transcription elongation factor, named DSIF (DRB sensitivity inducing factor) in human cells.
**SWI/SNF (switch/sucrose non-fermentable)**: a chromatin remodeling complex and founding member of a large group of DNA translocases.
**SYT9 (synaptotagmin 9)**: member of a large group of proteins that regulate membrane trafficking.
**TBP (TATA-binding protein)**: general transcription factor that binds specifically to the TATA box found about 30 base pairs upstream of the transcription start site in some eukaryotic promoters.
**TFIIH (general RNAPII transcription factor H)**: general transcription and repair factor required for both transcription and NER.
**UBR5 (ubiquitin protein ligase E3 component n-recognin5)**: ubiquitin ligase of the HECT family.
**USP7 (ubiquitin-specific protease 7)**: deubiquitylating enzyme with many substrates.
**Uvr (ultraviolet repair)**: several Uvr genes exist in *Escherichia coli*.
**UVSSA (UV-stimulated scaffold protein A)**: mutated in UV-sensitivity syndrome
**Xeroderma pigmentosum (XP)**: human repair disorder caused by mutations in XP-A–G and XPV.
