# Supplementary material for: The Cellular Response to Transcription-Blocking DNA Damage
Source: Trends Biochem Sci. 2018 May;43(5):327–41. doi: 10.1016/j.tibs.2018.02.010 (PMC5929563; doi:10.1016/j.tibs.2018.02.010)
Supplement: Supplementary file 1 [file mmc1.pdf]

Question 1:

Which kind of DNA damage is repaired by transcription-coupled nucleotide excision repair (TC-NER)?

❍ Double strand breaks

❍ Single stranded nicks in DNA

● Bulky DNA lesions blocking the progression of RNA polymerase

Explanation:

TC-NER recognizes bulky lesions in transcribed regions indirectly through stalled RNA polymerase

❍ UV-induced lesions in non-transcribed regions of the genome

Question 2:

The transcriptional response to UV irradiation in mammalian cells involves:

● Global decrease in transcriptional elongation rates

Explanation:

In response to UV transcriptional elongation rates are reduced dramatically and do not recovery fully until 12-48 hours after UV irradiation

❍ Complete removal of RNA polymerase from the gene body

❍ Global up-regulation of microRNAs

❍ Failure of RNA polymerase to terminate at transcriptional termination sites

Question 3:

Cockayne syndrome (caused by lack of functional CSB or CSA proteins) is characterized by:

● Photosensitivity and severe neurological symptoms

Explanation:

Cockayne syndrome is a complex disorder with patients displaying both photosensitivity and neurological symptoms

❍ Increased incidence of skin cancer

❍ Failure to shut down transcription after UV irradiation

❍ Increased ubiquitination of RNA polymerase

Question 4:

All of the following factors have been implicated in the global transcriptional shutdown and restart in mammalian cells following UV exposure, EXCEPT:

❍ Chromatin remodelers

❍ microRNA processing enzymes

❍ RNA-binding proteins

● DNA methyltransferases

Explanation:

DNA methyltransferases have not been implicated in transcriptional repression and restart after UV irradiation
